# Anti-Inflammatory Effects of a Methanol Extract from the Marine Sponge *Geodia cydonium* on the Human Breast Cancer MCF-7 Cell Line

**DOI:** 10.1155/2015/204975

**Published:** 2015-09-27

**Authors:** Susan Costantini, Giovanna Romano, Fabiola Rusolo, Francesca Capone, Eliana Guerriero, Giovanni Colonna, Adrianna Ianora, Gennaro Ciliberto, Maria Costantini

**Affiliations:** ^1^CROM, Istituto Nazionale Tumori “Fondazione G. Pascale”, IRCCS, 80131 Napoli, Italy; ^2^Department of Integrative Marine Ecology, Stazione Zoologica Anton Dohrn, Villa Comunale, 80121 Napoli, Italy; ^3^Servizio di Informatica Medica, Azienda Ospedaliera Universitaria, Seconda Università di Napoli, 80138 Napoli, Italy; ^4^Direzione Scientifica, Istituto Nazionale Tumori “Fondazione G. Pascale”, IRCCS, 80131 Napoli, Italy; ^5^Department of Biology and Evolution of Marine Organisms, Stazione Zoologica Anton Dohrn, Villa Comunale, 80121 Napoli, Italy

## Abstract

Many research groups are working to find new possible anti-inflammatory molecules, and marine sponges represent a rich source of biologically active compounds with pharmacological applications. In the present study, we tested different concentrations of the methanol extract from the marine sponge,* Geodia cydonium*, on normal human breast epithelial cells (MCF-10A) and human breast cancer cells (MCF-7). Our results show that this extract has no cytotoxic effects on both cell lines whereas it induces a decrease in levels of VEGF and five proinflammatory cytokines (CCL2, CXCL8, CXCL10, IFN-*γ*, and TNF-*α*) only in MCF-7 cells in a dose-dependent manner, thereby indicating an anti-inflammatory effect. Moreover, interactomic analysis suggests that all six cytokines are involved in a network and are connected with some HUB nodes such as NF-kB subunits and ESR1 (estrogen receptor 1). We also report a decrease in the expression of two NFKB1 and c-Rel subunits by RT-qPCR experiments only in MCF-7 cells after extract treatment, confirming NF-kB inactivation. These data highlight the potential of* G. cydonium* for future drug discovery against major diseases, such as breast cancer.

## 1. Introduction

Inflammation is a physiological process in response to acute tissue damage resulting from physical and ischemic injury, infection, exposure to toxins, chemical irritation, and/or other types of trauma. Many authors have suggested a correlation between chronic inflammation and cancer [1]. In fact, while inflammatory diseases increase the risk of developing many types of cancer, some nonsteroidal anti-inflammatory drugs reduce this risk for certain cancers (e.g., breast cancer) [2]. Interestingly, inflammation is involved in all three stages of tumor development, initiation, progression, and metastasis, where cytokines, chemokines, and growth factors play an important role in their evolution [3]. These are proteins that are expressed before and during the inflammatory process and play a key role during various disease stages so as to be considered as specific cancer markers as well as markers for various stages of the disease [2]. In general, the cytokinome is defined as the totality of these proteins and their interactions in and around cells [4]. Understanding the complex interaction network of cytokines in cancer patients should be very useful both to follow the evolution of cancer from its first steps and to define therapeutic strategies using innovative systems biology approaches. Several research groups are working to find new possible anti-inflammatory molecules [5]. Indeed, our laboratory has also recently evaluated the putative anti-inflammatory effects of different molecules such as sodium selenite, lipoic acid, and caffeic acid on cancer cell lines [6, 7] as well as of natural extracts from pomegranate (*Punica granatum* L.) seed oil [8] and* Juniperus oxycedrus* ssp.* oxycedrus* berries [9].

The marine environment, with its impressive biodiversity, is a rich natural resource of many biologically active compounds such as antioxidants, polyunsaturated fatty acids (PUFAs), sterols, proteins, polysaccharides, and pigments. In fact, many marine organisms live in complex habitats and are exposed to extreme conditions and produce a variety of biologically active secondary metabolites, which often have no terrestrial counterparts. Sponges seem to be particularly rich sources of bioactive compounds which show antiviral [10], antibacterial [11], and anti-inflammatory activities [12–15]. They are also promising candidates as anticancer drugs considering that 3 of the 4 commercial anticancer drugs available have been derived from marine sponges, including Ara-C from the marine sponge* Tectitethya crypta* [16] that is currently used in the routine treatment of patients with lymphoma, Halaven (eribulin) from the sponge* Halichondria okadai* used for the treatment of breast cancer [17], and Yondelis from the marine tunicate* Ecteinascidia turbinata* for the treatment of soft tissue carcinoma [18]. For example, several anti-inflammatory metabolites have been isolated from sponges belonging to the class of the Demospongiae, including cavernolide from* Fasciospongia cavernosa* [19], contignasterol from* Petrosia contignata* [20], and cyclolinteinone from* Cacospongia linteiformis* [21]. Therefore, the purpose of the present study was to investigate the potential effects of a methanol extract from the marine demosponge,* Geodia cydonium*, which has not been studied previously for drug discovery, on normal human mammary epithelial cells (MCF-10A) and cells of human breast cancer (MCF-7).

## 2. Materials and Methods

### 2.1. Sample Collection and Preparation of the Methanol Extract

The marine sponge* G. cydonium* (Porifera, Demospongiae, Astrophorida, Geodidae) was sampled in the “Parco Sommerso di Baia” (Gulf of Naples, Italy). The sample was collected in October 2013 at a depth of 20 metres. Individual specimens were placed separately into plastic bags and kept in seawater basins of the Marine Resources for Research Service at the Stazione Zoologica Anton Dohrn of Naples at a temperature of 15–20°C for one month.

Sponges were cut into small pieces and 10 g of tissue was ground into liquid nitrogen and then soaked in 200 mL of methanol for 2 hours under stirring. The solvent was removed and filtered through Whatman filter paper No. 1; the sponge tissue was squeezed in cheesecloth. The solvent was evaporated at low pressure using a rotavapor (Büchi Rotavapor R114) at 40°C and the residual aqueous extract was partitioned three times against ethyl acetate (2 : 1 v/v). To accelerate the phase separation, we centrifuged the extract at 2750 rcf for 10 minutes at 4°C and the organic phase was collected. The organic phase of various preparations was pooled and treated with ammonium sulfate to remove any aqueous residues. Nonpolar components and phenolic molecules were separated by this extraction procedure [22]. The final extract was dried and stored at −20°C until use.

### 2.2. Cell Culture

MCF-7 human breast cancer cells and MCF-10A normal human breast epithelial cells were cultured and expanded at 37°C in a humidified atmosphere of 5% CO_2_ in DMEM culture medium (Dulbecco's Modified Eagle's Medium, Lonza, Verviers, Belgium), supplemented with FBS (Invitrogen, Camarillo, CA, USA) at 10%, penicillin/streptomycin 1× (Euroclone, Devon, UK), and Glutamax 1× (Invitrogen). Moreover, in the case of MCF-10A the DMEM was supplemented also with human insulin (10 *μ*g/mL), epidermal growth factor (20 ng/mL), and hydrocortisone (0.5 *μ*g/mL) according to the procedure reported in [23].

### 2.3. Colorimetric Assay with Sulforhodamine B

Cell proliferation was assessed in the presence and absence of the methanol extract from* G. cydonium* by colorimetric assay with sulforhodamine B (SRB, Sigma Aldrich). The cells (3.5 × 10^−4^–4 × 10^−4^) were seeded in 96-multiwell plates in 200 *μ*L of culture medium and left to grow for 24 h at 37°C to allow adhesion. Cells were then treated with varying concentrations of the extract: 2 *μ*g, 50 *μ*g, and 200 *μ*g and incubated for 24 h. These concentrations were selected based on previous results on the effect of a methanol extract of this sponge on embryonic development in the sea urchin* Paracentrotus lividus*.

Dissolution of the extract was improved with 100 mM dimethyl sulfoxide (DMSO, Sigma Aldrich). From this stock solution, dilutions were made to obtain the different amounts of extracts with a final concentration of 0.05% DMSO. Control cultured cells were incubated with the same volume of the solvent. Cells were fixed by adding 10% trichloroacetic acid (Sigma Aldrich) for at least 1 h at 4°C. Subsequently, cells were washed with distilled water and air-dried. SRB (100 *μ*L) was added to each well and the plate was incubated for 30 min at room temperature in the dark. To remove excess dye, cells were washed with 1% acetic acid. The number of viable cells was directly proportional to the protein bound-dye formation which was then solubilized with 100 *μ*L of 10 mM Tris base solution pH 10.5, shaking the plates for at least 15 min on an orbital shaker to homogenize the dye solution. Optical density measurements were performed by using an automated 96-well plate reader (Microplate Reader, Bio-Rad, Hercules, CA, USA) at 540 nm. All experiments were performed in triplicate and were repeated for three times. Cellular viability was estimated as % compared to untreated cells.

### 2.4. Bio-Plex Assay

Levels of cytokines, chemokines, and growth factors were evaluated at the same time with the Bio-Plex assay. The multiplex biometric ELISA-based immunoassay, containing dyed microspheres conjugated with a monoclonal antibody highly specific for a target protein, was used, according to the manufacturer's instructions (Bio-Plex Bio-Rad), to evaluate the levels of different cytokines by Human Cytokine 27-Plex in MCF-10A and MCF-7 supernatants after treatment with increasing concentrations of sponge extract. In particular, the following cytokines were evaluated: IL-1*β*, IL-1ra, IL-2, IL-4, IL-5, IL-6, IL-7, CCL2, CCL11, CXCL10, CXCL8, IFN-*γ*, IL-9, IL-10, IL-12 (p70), IL-13, IL-15, IL-17, basic FGF, G-CSF, GM-CSF, MIP-1*α*, MIP-1*β*, PDGF-*ββ*, RANTES, TNF-*α*, and VEGF. Protein levels were determined using a Bio-Plex array reader (Luminex, Austin, TX, USA) that quantifies multiplex immunoassays in a 96-well format with very small fluid volumes. The analyte level was calculated using a standard curve, with software provided by the manufacturer (Bio-Plex Manager Software).

### 2.5. Bioinformatics Analysis

The expression levels of cytokines evaluated in MCF-10A and MCF-7 supernatants were compared by *t*-test. Values of *P* < 0.05 were considered to be statistically significant. The statistical program Prism 4 (GraphPad Software, San Diego, CA, USA) was used. The PANTHER (Protein ANalysis THrough Evolutionary Relationships) Classification System was designed to classify proteins according to their biological processes [24] as well as the metabolic pathways in which they are involved. Moreover, a network analysis was performed between the most significant proteins by ingenuity pathway analysis (IPA).

### 2.6. RNA Preparation and Reverse Transcription-qPCR (RT-qPCR) Analysis

Total RNA was extracted from MCF-7 cells that were either untreated (control) or exposed to increasing concentrations of sponge extract (2 *μ*g, 50 *μ*g, and 200 *μ*g) using the RNAeasy Mini Kit (Qiagen Inc., Valencia, CA, USA) according to the manufacturer's instructions. The extracted RNA was dissolved in diethylpyrocarbonate treated water, and its concentration and purity were assessed by measurement of optical density at 260/280 nm. RNA samples were quantified using a NanoDrop 2000 spectrophotometer (Thermo Scientific, Wilmington, DE). Two micrograms of total RNA of each sample was reverse-transcribed with SuperScript VILO cDNA Synthesis Kit (Life Technologies-Invitrogen, Carlsbad, CA, USA) according to the manufacturer's instructions and subsequently diluted with nuclease-free water (Life Technologies-Ambion).

Sequences for mRNAs from the nucleotide data bank (National Center for Biotechnology Information, USA) were used to design primer pairs for RT-qPCR (Primer Express, Applied Biosystems, CA, USA). Oligonucleotides were obtained from Sigma Aldrich. The primer sequences of 5 NF-*κ*B subunits' mRNAs are provided in Table 1. An appropriate region of 18S rRNA was used as control. RT-qPCR assays were run on a Step-One Real Time PCR System (Applied Biosystems). 2 *μ*L of cDNA was amplified in a total volume of 25 *μ*L containing 2X SYBR Green PCR Master Mix (Applied Biosystems) and 300 nM of forward and reverse primers. The thermal cycling conditions were as follows: 5 min of denaturation at 95°C followed by 44 cycles of a two-step program (denaturation at 95°C for 30 sec and annealing/extension at 60°C for 1 min). For each target, the primer sequences and the melting temperature are reported in Table 1. Dilutions of standards and test samples were run in duplicate. Each reaction was repeated at least three times. Expression levels of each target gene in MCF-7 cells treated with different concentrations of the sponge extract were compared with those in untreated cells using the REST tool (Relative Expression Software Tool, Weihenstephan, Germany) [25]. The REST mathematical model is based on the correction for exact PCR efficiencies and the mean crossing point deviation between sample group(s) and control group(s). Subsequently the expression ratio results of the investigated transcripts are tested for significance by a Pair Wise Fixed Reallocation Randomisation Test © and plotted using standard error (SE) estimation via a complex Taylor algorithm (http://www.gene-quantification.de/rest.html). Data were normalized using the 18S rRNA as housekeeping gene already used in our recent paper [26]. A 1 x-fold expression level was chosen as the threshold for significance of target genes.

## 3. Results and Discussion

### 3.1. Preparation and Viability Assay of the Methanol Extract

Sponges, due to the enormous diversity of associated microorganism communities, have always been considered an explicit source of pharmaceutical products. Our recent results showed that a diverse assemblage of bacteria resided in the marine sponge* G. cydonium* [27], including* Pseudoalteromonas* spp. that is reported to produce prodigiosin, a well-known tripyrrolic red pigment with immunosuppressive and anticancer activities, mainly in human breast cancer [28]. However, to our knowledge, this is the first study testing the biological activity of extracts of this sponge.

**Figure 1:**
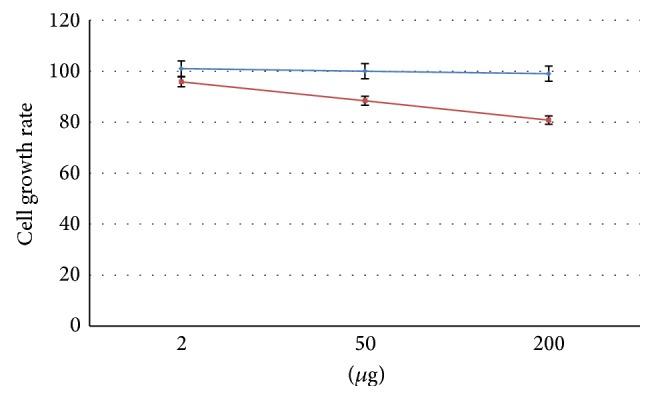
Cell growth rate after 24 h of treatment with different amount of sponge extracts in normal human breast epithelial cells, MCF-10A (blue), and human breast cancer cells, MCF-7 (red).

A methanol extract of* G. cydonium* was prepared as reported for other marine sponges [14, 15]. To identify the IC_50_ concentration, corresponding to the extract amount that causes 50% inhibition of cell growth, the cell viability of MCF-10A and MCF-7 was determined after 24 h stimulation with sponge extract by colorimetric assay with sulforhodamine B (Figure 1). We chose this incubation time because it has already been used in previous studies [14, 15]. Moreover, we tested the effect of methanol alone on two cell lines verifying that cell viability remained equal to 100%. Therefore, the cellular viability of untreated cells was used as control.

After 24 h of incubation, MCF-10A and MCF-7 cells retained a relatively constant viability with increasing concentrations of sponge extract, as demonstrated by the overlapping of growth curves in Figure 1. This suggests that our sponge extract had no antiproliferative or cytotoxic activities compared to other methanol extracts [14]. Interestingly, marine sponges of the genus* Geodia* (Demospongiae class, Tetractinellida order, Geodidae family) have been poorly explored for their chemical and biologically active components. Studies on the Mediterranean giant siliceous sponge* Geodia gigas* have led to the isolation of herbipoline (a biogenic amine) [29] and several steroidal ketones [30]. A series of cholestane derivatives have been isolated from another species,* G. megastrella* [31], and crude extracts of* G. corticostylifera* from the Brazilian coast have been reported as having antibacterial, antifungal, cytotoxic, haemolytic, and neurotoxic activities [32]. Some cyclic peptides geodiamolides A, B, H, and I were isolated from* G. corticostylifera* and their antiproliferative effects were demonstrated against sea urchin eggs and human breast cancer cell lines T47D and MCF7 by disorganizing actin filaments of these cells [33]. Furthermore, sponge depsipeptide, geodiamolide H, from* G. corticostylifera* increased gap junction length in hepatocarcinoma cells, affecting mainly the delivery pathway of connexin 43 that is membrane proteins that form gap junction channels between adjacent cells [34].

### 3.2. Evaluation of Cytokine Levels

Since some studies have reported that methanol extracts from marine sponges show anti-inflammatory effects [14, 15], we evaluated the concentrations of 27 cytokines (expressed in pg/mL) in MCF-10A and MCF-7 supernatants after treatment with* G. cydonium* sponge extract by multiplex biometric ELISA-based immunoassay. This simultaneous quantitative determination of a large cytokine panel enables us to correctly report ratios and dynamics between highly and poorly represented molecules. It represents an accurate, simple, specific, noninvasive, reproducible, and inexpensive method [6–8].

**Figure 2:**
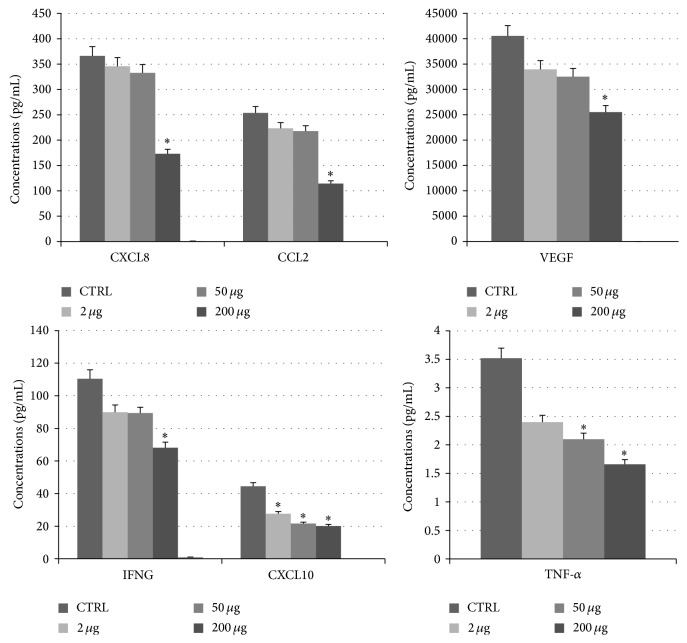
Statistically significant cytokine levels (expressed in pg/mL) evaluated in MCF-7 cells before and after treatment with different concentrations of sponge extract. Asterisks *∗* indicate the statistically significant differences between levels of untreated (used as controls and indicated with CTRL) and treated cells with *P* < 0.05 using the *t*-test. In the legend, we report the meaning of the bars by using different colors for untreated cells (CTRL) and three different treatments. In detail, we report CXCL8 (C-X-C motif ligand 8), CCL2 (C-C motif ligand 2), VEGF (vascular endothelial growth factor), IFNG (interferon gamma), CXCL10 (C-X-C motif ligand 10), and TNF-*α* (tumor necrosis factor alpha).

Results were compared with untreated cells used as controls. Experiments revealed that levels of VEGF and five proinflammatory cytokines (CXCL8, CXCL10, IFN-*γ*, CCL2, and TNF-*α*) decreased in a dose-dependent manner with increasing levels of sponge extract in breast cancer MCF-7 cells (Figure 2) but not in healthy breast MCF-10A cells. Some studies have already demonstrated that CXCL8 and VEGF promoters contain different recognition sites for nuclear factor- (NF-) kappa B [35]. For example, CCL2 expression is activated by NF-*κ*B [36], TNF-*α* induces the activation of antiapoptotic transcription factor NF-*κ*B [37], and IFN-*γ* requires NF-*κ*B to induce expression of the CXCL10 gene [38]. These data suggest a specific anti-inflammatory effect of the sponge extract on cancer cells.

Anti-inflammatory effects have already been reported in marine sponges, including cavernolide from* Fasciospongia cavernosa* [19], contignasterol from* Petrosia contignata* [20], and cyclolinteinone from* Cacospongia linteiformis* [21]. These effects can be explained by the inhibition of enzymatic activities such as inhibition of inducible nitric oxide synthase (iNOS), cyclooxygenase-2 (COX-2) gene expression, and plasma exudation* in vivo* in response to ovalbumin and prostaglandin E2. Another compound halipeptin A was isolated from the marine sponge* Haliclona* sp., which has shown anti-inflammatory activity on mouse paw edema assay [39]. This metabolite seems to be more potent compared to standard anti-inflammatory drugs like indomethacin and naproxen for anti-inflammatory effects. Another metabolite petrosaspongiolide M, isolated from the Caledonian marine sponge* Petrosaspongia nigra*, is a potent inhibitor* in vivo* and* in vitro* of phospholipase A2 (PLA2), demonstrating anti-inflammatory activity in models of acute and chronic inflammation [40].

However, there are few reports of sponge-derived compounds acting on specific cytokines. For example, barettin isolated from the marine sponge* Geodia barretti* was able to inhibit the secretion of two proinflammatory cytokines, IL-1*β* and TNF*α*, from LPS-stimulated THP-1 cells, and the combination of its anti-inflammatory and antioxidant activities suggests that this compound could have an atheroprotective effect [41]. Moreover, Pfeifer et al. (1992) [42] identified a tumor necrosis-like factor in the sponge* G. cydonium*, an endotoxin that mediates necrosis formation in xenografts.

Our study provides a complete evaluation of the anti-inflammatory effects of a methanol extract from the marine sponge* G. cydonium*, using a cytokinome approach where the cytokinome is defined as the totality of the cytokines and their interactions in and around biological cells as in [4]. Understanding the complex interaction network of cytokines should be very useful both to follow the evolution of cancer from its early steps and to define innovative therapeutic strategies by using systems biology approaches [2, 4]. However, a correct and comprehensive understanding of cytokine functions can be obtained only from simultaneous and coherent measurements of the serum concentrations of cytokines. This point raises the inherent difficulty of a simultaneous measurement of the cytokine concentrations to obtain correct internal ratios among the various molecules present in the same biological fluid or cellular supernatants due to the often large difference in concentrations spanning several orders of magnitude. At present, it is possible to effectively characterize cytokine levels only using a broad-spectrum bead based multiplex immunoassay that was used in [4] and the present study.

### 3.3. Functional and Network Analysis

**Figure 3:**
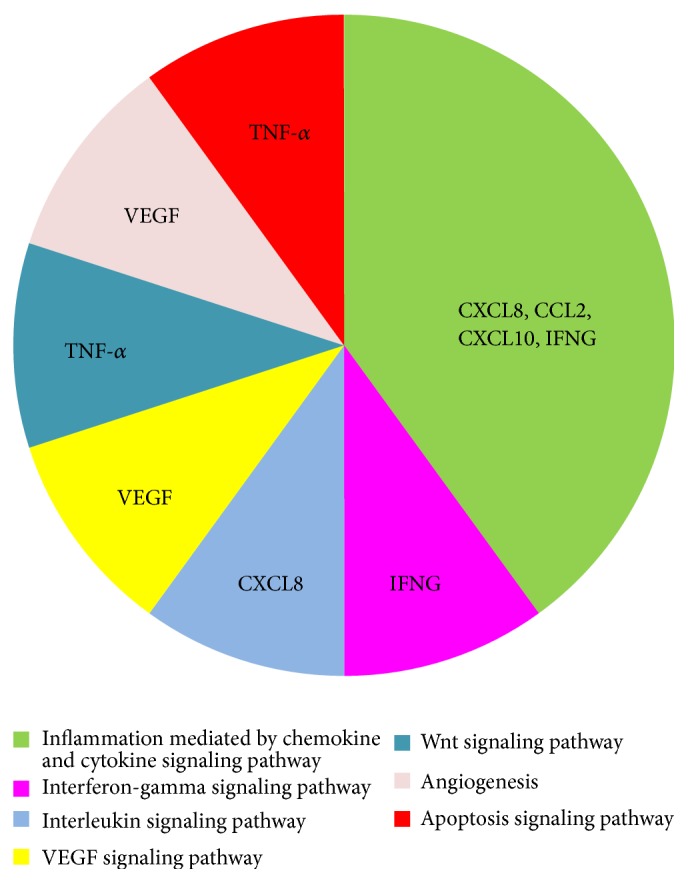
PANTHER pathway analysis in which we show the pathways related to the following six significant proteins: CXCL8 (C-X-C motif ligand 8), CCL2 (C-C motif ligand 2), VEGF (vascular endothelial growth factor), IFNG (interferon gamma), CXCL10 (C-X-C motif ligand 10), and TNF-*α* (tumor necrosis factor alpha).

To verify if these proteins are correlated with one another through NF-*κ*B, we performed a functional analysis on CCL2, CXCL8, CXCL10, IFN-*γ*, TNF-*α*, and VEGF using the PANTHER tool [24] and then conducted a network study by ingenuity pathway analysis (IPA).  Figure 3 shows that these six proteins are involved in 7 metabolic pathways: CCL2, CXCL8, CXCL10, and IFN-*γ* in the inflammation signaling pathway mediated by chemokines and cytokines, VEGF in angiogenesis and the VEGF signaling pathway, TNF-*α* in apoptosis and the Wnt signaling pathways, CXCL8 in the interleukin signaling pathway, and IFN-*γ* in the interferon-gamma signaling pathway.

**Figure 4:**
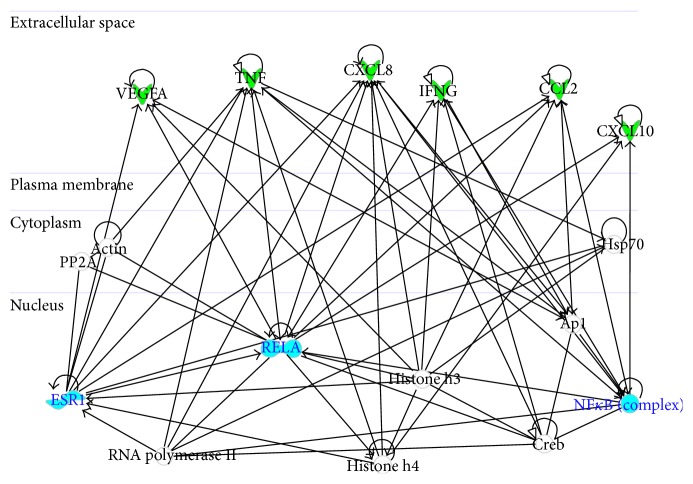
Interactomic analysis by ingenuity pathway analysis (IPA) of significant molecules. The interactome shows the close functional association between significant cytokines (indicated with green symbols) as well as the paths in which other functionally relevant molecules are also involved (indicated with white symbols). In detail, the cytokines reported are CXCL8 (C-X-C motif ligand 8), CCL2 (C-C motif ligand 2), VEGF (vascular endothelial growth factor), IFNG (interferon gamma), CXCL10 (C-X-C motif ligand 10), and TNF (tumor necrosis factor alpha). Moreover, three HUB nodes such as ESR1 (estrogen receptor 1), NF-*κ*B (nuclear factor kappa B), and RELA (v-rel avian reticuloendotheliosis viral oncogene homolog A) subunits are indicated by cyan symbols.

Moreover, interactomic analysis shows that all of the six analyzed significant cytokines are involved in a network named “*Cellular Movement, Immune Cell Trafficking, Hematological System Development and Function*” on the basis of associated functions and data mining from experimental studies reported in the literature (Figure 4). This network reveals that these proteins are connected with some HUB nodes such as NF-*κ*B subunits (NF*κ*B complex and RELA) and ESR1 (estrogen receptor 1), which is a commonly used clinical breast cancer marker [43]. More specifically, (i) CCL2, CXCL8, CXCL10, IFN-*γ*, TNF-*α*, and VEGF are connected with NF-*κ*B subunits (NF*κ*B complex and RELA) and (ii) CCL2, CXCL8, TNF-*α*, and VEGF with ESR1.

Therefore, our interactomic studies confirmed that these six significant cytokines were mutually interconnected by the NF-*κ*B complex and suggested that a decrease in the levels of these cytokines can inhibit the activation of NF-*κ*B by blocking tumor growth.

### 3.4. Expression Levels of NF-*κ*B Subunits in MCF-7 Cells

It is known that the NF-*κ*B family shares a Rel homology domain in their N-terminus. In particular, a subfamily of NF-*κ*B proteins, including RELA, RELB, and c-Rel, has a transactivation domain in their C-terminal regions. On the other hand, NFKB1 and NFKB2 proteins are synthesized as large precursors (p105 and p100), which are processed to generate the mature NF-*κ*B subunits (p50 and p52), respectively [44].

**Figure 5:**
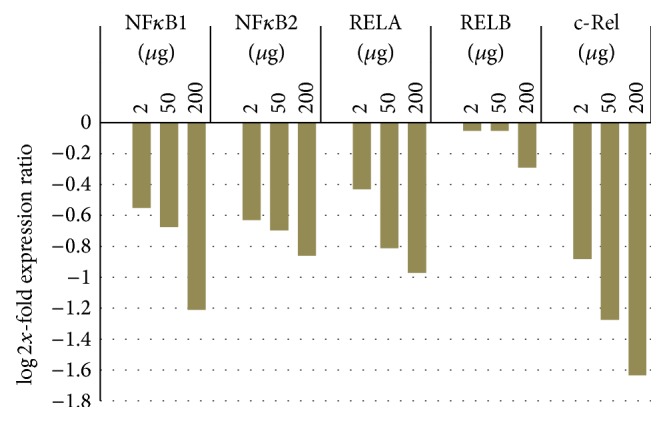
Changes in the expression levels of five NF-*κ*B subunits, NFKB1 (nuclear factor kappa B1), NFKB2 (nuclear factor kappa B2), RELA (v-rel avian reticuloendotheliosis viral oncogene homolog A), RELB (v-rel avian reticuloendotheliosis viral oncogene homolog B), and c-Rel (cellular counterpart of the v-Rel oncogene) in breast cancer MCF-7 cells treated with increasing concentrations (2 *μ*g, 50 *μ*g, and 200 *μ*g) of sponge extract compared to those of untreated cells.

Therefore, to verify if NF-*κ*B was modulated by the sponge extract, the gene expression levels of the five NF-*κ*B subunits, NFKB1, NFKB2, RELA, RELB, and c-Rel, in MCF-7 cells, treated with increasing concentrations (2 *μ*g, 50 *μ*g, and 200 *μ*g) of sponge extract, were evaluated by RT-qPCR compared to those in untreated cells (Table 1). No significant changes in expression levels were observed for NFKB2, RELA, and RELB, whereas the two genes, NFKB1 and c-Rel, showed a statistically significant downregulation after treatment with sponge extract (Figure 5), thus confirming the downregulation of NF-*κ*B. We are currently performing Western blot analysis to validate these results by the same protocol recently used in our group [45]. Preliminary results indicate no significant changes for RELA expression and a significant downregulation for NFKB1 in MCF-7 cells after treatment with 200 *μ*g of sponge extract compared to those in untreated cells (see Figure  1S in Supplementary Material available online at http://dx.doi.org/10.1155/2015/204975).

## 4. Conclusions

Cell response to stress includes a wide range of molecular processes that are activated through the modulation of signaling pathways that lead to repair, adaption, or cell death. Moreover, stress may induce a prolonged inflammation leading to several pathological conditions, which include cancer [3]. For this reason, we are working to discover novel anti-inflammatory molecules [6–8].

In the past decades, several studies have shown that marine sponges contain compounds with anti-inflammatory activities [14, 15]. Our results show that* G. cydonium* sponge extract (i) does not have a cytotoxic effect on both MCF-10A and MCF-7 cell lines, (ii) induces a decrease in VEGF levels and of five proinflammatory cytokines (CCL2, CXCL8, CXCL10, IFN-*γ*, and TNF-*α*) in a dose-dependent manner only in MCF-7 cells, and (iii) induces the downregulation of two NF-*κ*B subunits, NFKB1 and c-Rel.

In conclusion, our findings reveal for the first time that a methanol extract from the marine sponge* G. cydonium* had no antiproliferative or cytotoxic activities but anti-inflammatory effects by cytokinome approach on the human breast cancer MCF-7 cell line.

Studies are currently underway to chemically characterize the compounds produced by* G. cydonium* that are responsible for the effects on breast cancer cell lines for future drug development. Extension of this approach may help elucidate the function of individual molecular species and prioritize them as drug targets.

## Supplementary Material

Supplementary Material: Representation of the intensities of the bands associated to NFKB1 and RELA obtained by Western Blotting in MCF-7 cells after treatment with sponge extract.

## Figures and Tables

**Table 1:** Parameters for RT-qPCR analysis.

Gene	Tm [°C]	Sequence (5′→3′)
NFKB1	59	CCTCTGTGTTTGTCCAGCT (19)
		CCGAAAAATTGGGCATGAGC (20)

NFKB2	58	GCTTCTCTGCCTTCCTTAG (19)
		CACAGAGCCTGCTGTCTTG (19)

RELA	58	CACGAGCTTGTAGGAAAGG (19)
		GCGCTGACTGATAGCCTG (18)

RELB	60	TGCTTCGGTCTGGGCCAG (18)
		CAATTCATCTGTGCTCCTGG (20)

C-Rel	60	TCCTGACTGACTGACTGCG (19)
		CTAAAACGCATTCCCCTCTG (20)

18S	60	CTGCCCTATCAACTTTCGTG (20)
		GTAGTTTCTCAGGCTCCCTCTC (22)
