# Supplementary material for: Anti-Inflammatory Effects of a Methanol Extract from the Marine Sponge Geodia cydonium on the Human Breast Cancer MCF-7 Cell Line
Source: Mediators Inflamm. 2015 Sep 27;2015:204975. doi: 10.1155/2015/204975 (PMC4600500; doi:10.1155/2015/204975)
Supplement: Supplementary file 1 — Supplementary Material: Representation of the intensities of the bands associated to NFKB1 and RELA obtained by Western Blotting in MCF-7 cells after treatment with sponge extract. [file 204975.f1.doc]

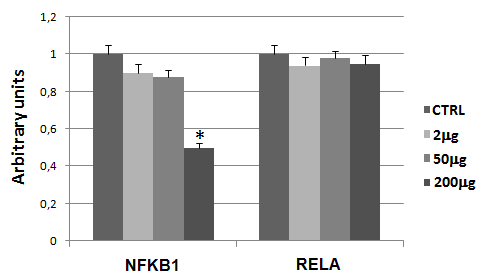


**Figure S1.** Representation of the intensities of the bands associated to NFKB1 and RELA obtained by Western Blotting performed using the protocol used recently in our group [33], rabbit anti NFkB p105/p50 (NFKB1) and p65 (RELA) antibodies (ABCAM, Cambridge) and γ-tubulin (Santa Cruz Biotechnology, Santa Cruz, CA, USA) to ensure the equal loading of samples in each lane. The bands were scanned with a laser scanner (Richo MP C3000) and their intensities were quantified with Image J software (NIH) and expressed as arbitrary units when compared to those of the untreated cells. The error bars shown in the histograms represent the standard deviation from the mean of different densitometric scanning in three experiments. Also, we inserted an asterisk * when the difference between the intensities in untreated and treated cells was statistically significant by T-test (with p-value <0.05).
